# Role of ADAM and ADAMTS metalloproteinases in airway diseases

**DOI:** 10.1186/1465-9921-10-127

**Published:** 2009-12-24

**Authors:** Genevieve Paulissen, Natacha Rocks, Maud M Gueders, Celine Crahay, Florence Quesada-Calvo, Sandrine Bekaert, Jonathan Hacha, Mehdi El Hour, Jean-Michel Foidart, Agnes Noel, Didier D Cataldo

**Affiliations:** 1 Laboratory of Tumor and Development Biology, Groupe Interdisciplinaire de Génoprotéomique Appliquée- GIGA, University of Liège and CHU of Liège, Sart-Tilman, Belgium

## Abstract

Lungs are exposed to the outside environment and therefore to toxic and infectious agents or allergens. This may lead to permanent activation of innate immune response elements. **A D**isintegrin **A**nd **M**etalloproteinases (ADAMs) and ADAMs with **T**hrombo****s****pondin motifs (ADAMTS) are proteinases closely related to Matrix Metalloproteinases (**MMP**s). These multifaceted molecules bear metalloproteinase and disintegrin domains endowing them with features of both proteinases and adhesion molecules. Proteinases of the ADAM family are associated to various physiological and pathological processes and display a wide spectrum of biological effects encompassing cell fusion, cell adhesion, "shedding process", cleavage of various substrates from the extracellular matrix, growth factors or cytokines... This review will focus on the putative roles of ADAM/ADAMTS proteinases in airway diseases such as asthma and COPD.

## Introduction

The lung is continuously exposed to the outside environment and various potential aggressions such as noxious and infectious agents or allergens. The innate immune responses are permanently activated in this particular organ. Moreover, secretory materials such as surfactant and mucous also contribute to host defense against inflammation. Among airway diseases, asthma and COPD (**C**hronic **O**bstructive **P**ulmonary **D**isease) appear to be growing public health concerns worldwide and the number of listed asthmatic and COPD patients still increases over time.

Asthma is a complex clinically-defined syndrome mainly characterized by symptoms (wheezing, cough, breathlessness) and airway obstruction. Hallmarks of asthma are mainly airway hyperresponsiveness caused by a wide variety of stimuli and airway inflammation involving eosinophils and mast cells. Moreover, an asthma-associated remodeling of the airways including extensive changes in the extracellular matrix has been characterized. The main changes reported are a subepithelial fibrosis, a smooth muscle hypertrophy, a glandular metaplasia in the bronchial epithelium, and the deposition of extracellular matrix components throughout the airway wall. These features are very often associated with altered behaviour of airway structural cells including epithelial cells or fibroblasts [1,2].

COPD is characterized by a progressive airway obstruction mainly linked to tobacco consumption and/or toxic fumes and other environmental factors. COPD patients also display profound modifications of the extracellular matrix leading to an airway remodeling including collagen fibers deposition in the bronchial and bronchiolar walls, mucous hyperplasia, and smooth muscle cell hypertrophy [3-5].

As the key role of extracellular matrix and soluble mediators has been unveiled, there is accumulating evidences demonstrating the crucial role played by matrix metalloproteinases (MMPs) in lung diseases [6,7]. These aspects have been largely discussed in previous reviews [8,9]. The present review focuses on another subfamily of proteinases also belonging to the metzincins (zinc-bearing proteinases) and structurally related to MMPs: the ADAMs (**A D**isintegrin **A**nd **M**etalloproteinase) [10-14]. ADAM proteinases have been described as "signalling scissors" since they are associated to shedding processes of key factors implicated in physiological as well as in pathological activities [15]. This shedding process is quite interesting as it appears as an emerging concept that could be implicated in airway diseases. Indeed, ADAM-17 has been defined as the prototypical TNF-α convertase enzyme [16]. Besides this very well known example, many other sheddase activities have been reported and can address many physiological processes such as the regulation of cell proliferation by cleavage of membrane-bound heparin-binding epidermal growth factor (HB-EGF) [17]. Some cell receptors including the low-affinity immunoglobulin E receptor (CD23) can also be targeted by sheddases. Indeed, ADAM-10 appears to be the main sheddase for CD23 leading to increased levels of its soluble form [18,19]. The literature emerging in the last years suggests that ADAMs scissors-function plays a crucial role in airway diseases.

In the present review, after a brief general description of ADAM proteins, we discuss the implications of these proteinases in various physiological and pathological processes. The potential contribution of ADAM/ADAMTS proteins to asthma pathology will be described as well as ADAMs/ADAMTS' involvement in COPD.

## Structural features of ADAMs

To date, about 40 members of the ADAM family have been described in different species (for a constantly updated database, see http://people.virginia.edu/~jw7g/Table_of_the_ADAMs.html and http://degradome.uniovi.es/). Twenty-five ADAMs are expressed in *Homo sapiens *while thirty-five members are expressed in *Mus musculus*. Together with ADAMTS (ADAMs with **T**hrombo**s**pondin motifs type I) and SVMPs (**S**nake **V**enom **M**etallo**p**roteinases), ADAM proteinases constitute the subfamily of adamalysins [12] which belongs to the superfamily of metzincins. This superfamily also includes astacins, matrixins (also referred to as matrix metalloproteinases), serralysins and pappalysins [20,21]. Those metzincins are characterized by *(1) *a catalytic site containing a consensus sequence (HEXXHXXGXXH) in which three histidine residues coordinate a zinc ion and *(2) *by a conserved methionine residue forming a "Met-turn" beneath the active zinc site. This "Met-turn" provides a hydrophobic environment for the zinc ion and the three ligating histidine residues at the catalytic centre of the enzyme [22,23].

**Figure 1:**
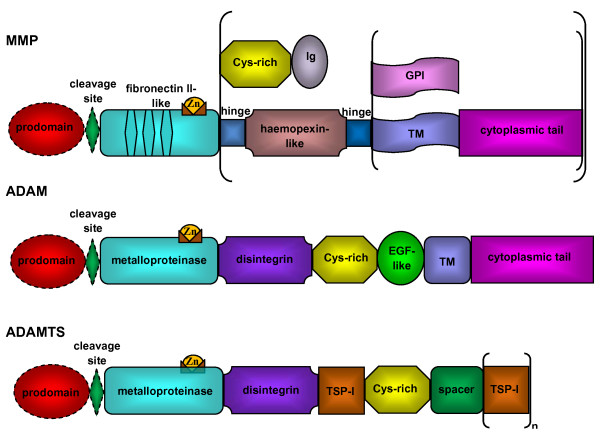
**Structural organization of MMPs, ADAMs and ADAMTS**. The typical structure of MMP is made of a prodomain, a furin cleavage site (all MT-MMPs, MMP-21,-23, and -28), a catalytic metalloproteinase domain with fibronectin type II repeats (MMP-2, MMP-9), a linker peptide and a haemopexin domain (except for MMP-7, -26, and -23), a linker peptide, a transmembrane domain and cytoplasmic tail (MMP-14, -15, -16, -24) or glycosylphosphatidylinositol (GPI) anchor (MMP-17, -25). MMP-23 bears C-terminal cysteine-rich (Cys-rich) and Ig-like (Ig) domains and its propeptide lacks a cystein switch motif. Common structure of ADAMs is a prodomain, a cleavage site (by a furin or furin-like proprotein convertase except for ADAM-8 and ADAM-28 which use an autocatalytic process), a metalloproteinase domain, a disintegrin domain, a cysteine-rich region (Cys-rich), an epidermal-growth factor repeat (EGF-like), a transmembrane domain (TM) and a cytoplasmic tail. ADAMTS do not possess a transmembrane domain (TM) but bear a various number of thrombospondin type I motifs (TSP-1) at their C-terminal extremity.

Structure of ADAMs and ADAMTS is highly conserved and involves metalloproteinase and disintegrin domains endowing them with features of both proteinases and adhesion molecules [11,13]. As illustrated in figure 1, the detailed structure of ADAMs is far more complex than that of MMPs. Domains shared with MMPs are the prodomain maintaining the catalytic site inactive and the metalloproteinase domain containing the Zinc binding site. ADAM activation mechanisms are mostly similar to MMP's activation and generally imply the prodomain removal from the precursor protein *via *a proprotein convertase of furin type [24]. However, maturation of some ADAMs, such as ADAM-8 and ADAM-28 occurs as an autocatalytic process [25,26]. The metalloproteinase domain with its catalytic consensus site is active in only about half of ADAM proteinases. The following domains are characteristic of ADAMs and include a disintegrin domain mediating cell-cell, cell-matrix interactions via the interaction with integrins; a cystein-rich domain implicated in cell adhesion; an epidermal growth factor (EGF)-like domain and a cytoplasmic tail involved in various intracellular signalization pathways [11].

Although the structure of ADAM and ADAMTS proteinases is closely related, ADAMTS molecules are characterized by a various number of thrombospondin type one motifs (TSP-1) at their C-terminal end and the absence of transmembrane and cytoplasmic domains [10,27] (figure 1). In the C-terminal extremity, different types of modules have been described for some of the ADAMTS. All these data are regularly updated on http://www.lerner.ccf.org/bme/apte/adamts.

The metalloproteinase system is controlled by endogenous physiological inhibitors ("**T**issue **I**nhibitors of **M**etallo**p**roteinases" or TIMPs) which are small proteins with molecular weights ranging from 21 to 28 kDa. These inhibitors display six disulfide bonds in their structure forming a rigid conformation which is mandatory for their biological activity. TIMPs are able to inhibit proteinase activity of several members of the ADAM family [28-31]. N-terminal domain of TIMPs and more specifically the "functional binding edge" is interacting with the catalytic domain of the ADAM proteinase [32]. The interaction of the catalytic site-bound Zinc atom with a cystein present in the N-terminal extremity of TIMP leads to an inactivation of ADAMs. This process has been described for ADAM-17 or ADAMTS-4 interacting with TIMP-3 [30,32]. More recently, a novel method of purification using sodium chlorate has confirmed that C-terminal domain of ADAMTS-4 and -5 and more particularly their TS-domains favors the interaction with the N-terminal domain of TIMP-3 [33].

When taking into consideration the complex multi-domain structure of ADAMs and ADAMTS, one can anticipate their implication in many physiological and pathological processes. From these complex structural features and bearing in mind that only half of proteins of this family display a catalytic activity, one can expect that functions of ADAMs and ADAMTS will not be restricted to the cleavage of extracellular matrix or mediators but will embrace various functions including the regulation of cell-cell and cell-matrix interactions. Although these ADAM/ADAMTS functions are not yet as much discerned as those of MMPs, a real interest from the scientific community has emerged these last years, specifying not only the exact structure of these proteins, but also identifying new features involving ADAMs in health and diseases. In this review, we are discussing known and potential implications of ADAMs in lung homeostasis as well as in its deregulation.

## Implication of ADAMs and ADAMTS in physiological and pathological processes

Since ADAM proteinases are defined as multi-domain proteins, studies have focused their attention on the multiple functions that can be ascribed to these proteinases. ADAMs have been described in various physiological processes such as egg fertilization, myogenesis, cell fate determination but also in diverse pathological processes.

### Physiological processes

Properties attributed to ADAMs are evidently crucial when one considers their structural organization. We will present hereafter selected examples illustrating the diversity of biological processes that can be affected by ADAM proteins. Most of ADAMs are membrane-bound proteins and can assist cell fusion, cell adhesion, peptidic mediators processing, linked or not to plasma membrane. They also play a key role in some intracellular signaling pathways. The final picture is rendered even more complex since alternative splicing can induce variations in the C-terminal region of membrane-bound ADAMs and thereby give rise to different cytosolic tails or secreted proteins [34].

Some ADAMs appear essential in cell fusion processes. It is worth underlining that the two first identified ADAMs (ADAM-1 and -2) were recognized as fertilin-alpha and -beta in 1987 [35] since they could induce the fusion of the sperm with the egg. This process is mediated through the interaction of the disintegrin domain of ADAM-2 present on the sperm with integrin α6β1 beared on the egg surface [36]. Moreover, ADAM proteins are key enzymes in embryonic development since ADAM-10 is able to cleave NOTCH protein and consequently regulate central nervous system development [37]. ADAMs also contribute to intracellular signalling processes and have the ability to interact with tyrosine kinases and some components of the cytoskeleton through their cytoplasmic domain [11]. The disintegrin domain of some ADAMs is able to regulate cell adhesion through interaction with various integrins. For instance ADAM-15 is described as a novel component of adherens junctions [38]. Importantly, as stated earlier, ADAMs/ADAMTS are able to cleave membrane-bound growth factors, cytokines and proteoglycans, leading to the detachment of mature soluble forms. This process is largely referred to as *sheddase *activity. So far, the most studied sheddases are ADAM-17 and ADAM-10 responsible for the cleavage of pro-TNF and CD23, respectively [16,18,39]*. *ADAMTS proteinases also display a catalytic activity. Indeed, ADAMTS-4 and ADAMTS-5 are able to cleave aggrecan [40,41], ADAMTS-2 processes type I, II and III procollagen chains [42].

### Pathological processes

Dysregulation of ADAMs expression has been reported in different types of pathologies such as cancer, osteoarthritis, neurodegenerative inflammation or asthma. In most studies, an overexpression of these proteinases has been described and is linked to a dysregulation of tissue homeostasis sometimes leading to a specific pathological phenotype. ADAMs might therefore be considered as potential candidates to target in a therapeutic setting. For instance, ADAM-17 expression is increased in breast cancer tissues and its expression is higher in advanced-grade than low-grade tumors. Patients displaying a huge expression of this proteinase have a shorter overall survival than those with a low expression of ADAM-17 [43] suggesting that ADAM-17 might be a good target to predict the outcome of cancer development. ADAMTS-4 and ADAMTS-5 are implicated in osteoarthritis development since ADAMTS-4/-5 double-knockout animals are less affected than wild-type mice [44,45]. Alzheimer's disease is characterized by beta amyloid deposition in the brain. ADAM-10 acts as an alpha-secretase and thereby cleaves the amyloid precursor to release a soluble component. Many authors have hypothesized that overexpression of ADAM-10 might have beneficial effects on the pathological deposition of amyloid protein [46,47] since ADAM-10 overexpressing mice display reduced susceptibility to amyloid deposition [47].

The complex structure of ADAMs also suggests that these enzymes may be functionally relevant to different steps linked to asthma pathogenesis. Indeed, the active metalloproteinase domain of some ADAMs might be important to shed growth factors and cytokines, contributing in this way to the control of inflammation which is a hallmark of asthma pathology. Disintegrin domain might also act in concert with cystein-rich region to interfere with pro-inflammatory cytokines [48].

These data illustrate how much ADAMs are multifunctional proteins and suggest that these proteinases may serve as mediators during the progression of asthmatic pathology but also COPD (table 1).

**Table 1:** ADAMs/ADAMTS modulation in airway diseases.

ADAMs	Modulation	Type of airway disease	Type of study	Reference
**ADAM-8**	↗	asthma	human	[51,57,74]
	
	↗	asthma	mouse	[58-60,73]*[Paulissen et al, submitted]*

**ADAM-9**	↗	asthma	human	[51]

**ADAM-10**	↗	asthma	mouse	[73]

**ADAM-12**	↗	asthma	human	[51]

**ADAM-17**	↗	asthma	mouse	[73]
	
	↗	COPD	rat	[80]

**ADAM-28**	↗	asthma	mouse	[73]

**ADAM33**	↗	asthma	human	[57,71,82]
	
	SNP	COPD	human	[79,83]

**ADAMTS-1**	↘	asthma	human	[51]

**ADAMTS-12**	SNP	asthma	human	[84]

**ADAMTS-15**	↘	asthma	human	[51]
	
	↗	asthma	mouse	[73]

SNP: Single Nucleotide Polymorphism

## Expression of ADAMs and ADAMTS in the lung

In the lung, different cell types can express different classes of proteinases. Some structural cells from bronchial tree are able to produce enzymes belonging to the metzincin superfamily that are important in regulation processes and in the cascade leading to inflammation. However, some data - especially those concerning the expression of ADAMs in lung tissues - are more recent and rather incomplete [49] (table 2). In lung tissue, an expression of ADAM-8, -9, -10, -12, -15, -17 and ADAMTS-1, -2, and TS-12 has been observed [50] with a modulation of ADAM-12 and ADAMTS-1 in tumors [50]. In sputum cells, an expression of ADAM-8, -9, -10, -12, -15, -17 and ADAMTS-1, TS-15 has been reported [51]. Moreover, epithelial cells have been shown to express ADAM-9, -10, -12, -15, -17 and ADAMTS-1 with an exception for immortalized bronchial epithelial cells (BEAS-2B) which do not express ADAM-12 [50]. Another epithelial cell line (A549, an alveolar epithelial cell line) was shown to express ADAM-19 and ADAMTS-9 [52]. Whereas mesenchymal cells such as fibroblasts and smooth muscle cells abundantly express ADAM-33 [53-55], epithelial cells may also express this proteinase [49,56]. Although only some authors have reported that airway epithelial cells express ADAM-8 [57,58], all authors have agreed to confirm that inflammatory cells produce ADAM-8, a proteinase that has been suggested to be a key mediator in inflammatory processes [51,57-60].

**Table 2:** ADAMs/ADAMTS expression in lung cell types.

ADAMs	Lung cell types	Reference
**ADAM-8**	epithelial cells	[49,57,58]
	inflammatory cells	[51,57,59]*[Paulissen et al, submitted]*
	smooth muscle cells	[57]*(-) *[58]*(+)*

**ADAM-9**	epithelial cells	[49,85]
	inflammatory cells	[49]
	smooth muscle cells	[49]

**ADAM-10**	epithelial cells	[49]
	smooth muscle cells	[49]

**ADAM-12**	inflammatory cells	[50]
	smooth muscle cells	[50]

**ADAM-17**	epithelial cells	[49,86]
	inflammatory cells	[49]
	smooth muscle cells	[49]

**ADAM-19**	epithelial cells	[49,52]
	endothelial cells	[49]
	inflammatory cells	[49]
	smooth muscle cells	[49]

**ADAM-28**	epithelial cells	[87]

**ADAM-33**	epithelial cells	[57]*(+) *[88]*(-)*
	endothelial cells	[49]
	fibroblasts	[54,88]
	inflammatory cells	[49,57]
	smooth muscle cells	[55,57,82,88]

## Contribution of ADAM and ADAMTS proteinases to the asthmatic phenotype

In some individuals, an inflammatory reaction occurs in the lungs after exposure to specific allergens. Following a single allergen exposure, an early-phase reaction is produced in pulmonary tissues followed by a late-phase reaction. The early-phase reaction is characterized by the activation of mast cells and macrophages and the release of various mediators including histamine and eicosanoids while the late-phase reaction consists of recruitment of eosinophils, CD4^+ ^T cells, basophils and neutrophils. Moreover, T helper cells amplify the inflammatory response via the release of Th_2 _cytokines. Following repetitive exposure to allergens, a chronic inflammation develops with associated tissue alterations such as mucus hypersecretion, vascular leakage, smooth muscle contraction, and bronchial hyperresponsiveness [61]. Asthma is associated to an airway remodeling that includes 1) a subepithelial fibrosis which appears as a pathognomonic feature of asthmatic bronchi, 2) changes in extracellular matrix composition with an absence of classical components of basement membrane (mainly collagen IV and laminin) and a fragmentation of elastic fibers, 3) a goblet cells hyperplasia and 4) increased angiogenesis [62].

**Figure 2:**
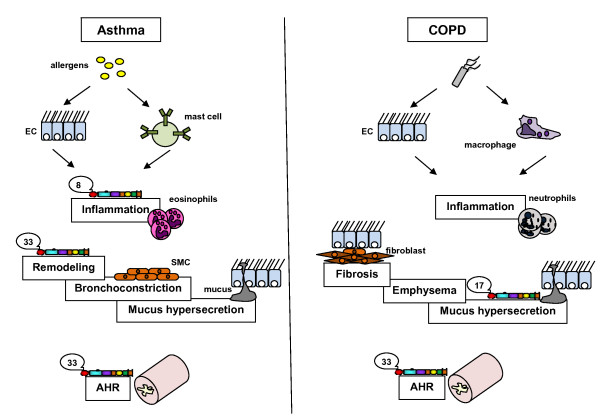
**Intervention of ADAM/ADAMTS proteinases in asthma and COPD**. Succinctly, in asthma, inhaled allergens provoke the degranulation of sensitized mast cells and the activation of epithelial cells (EC) while in COPD, inhaled cigarette smoke activates epithelial cells and macrophages. After a first challenge in both diseases, an inflammatory reaction occurs resulting in the recruitment of eosinophils and CD4^+ ^T cells for asthma, neutrophils and CD8^+ ^T cells for COPD. Following a chronic inflammation, tissue alterations such as mucus hypersecretion, bronchoconstriction appear in asthma while small airway fibrosis, alveolar destruction (emphysema) and mucus hypersecretion occur in COPD. An airway hyperresponsiveness is linked to both diseases. However, it is reversible in asthma but not in COPD. ADAM-8 plays a role in asthma-related inflammation while ADAM-33 is associated to remodeling processes and hyperresponsiveness associated to asthma. In COPD, ADAM-17 acts on mucus hypersecretion process while ADAM-33 is associated with COPD-related hyperresponsiveness.

Over the last years, the attention has risen about the roles of ADAM proteinases in processes leading to the asthmatic phenotype described above (see figure 2). ADAM-33 was one of the first ADAM proteinases to be identified as an asthma susceptibility gene after an ambitious study based on a vast genome screening [53]. An association of ADAM-33 gene polymorphism with the hyperresponsiveness linked to the asthmatic pathology has now been confirmed by many studies [63-66]. However, these data need to be clarified since not all authors report such a link between asthma and ADAM-33 [67,68]. These studies linking asthma and variants in ADAM-33 gene are summarized in table 3. Discrepancies between published studies can be explained by the diversity of studied populations and the complexity of this gene subject to alternative splicing processes. Moreover, important differences of statistical power of all these studies might also account for some of the reported differences between cohorts. Molecular mechanisms and exact roles of ADAM-33 in the pathological process leading to asthma are therefore not yet fully elucidated. While it was reported that ADAM-33 expression is mainly detectable in smooth muscle cells and in fibroblasts, authors have recently shown that ADAM-33 is also expressed by other cell types including endothelial cells [49,69]. ADAM-33 therefore might play a key role in asthma-associated airway remodeling since the purified catalytic domain of this proteinase provokes an increased development of the vascular network in asthmatic patients [70]. An argument to speculate for a possible key role of ADAM-33 in asthma physiopathology is the increased ADAM-33 expression reported after stimulation by some Th_2 _cytokines (IL-4 and IL-13) [71]. In humans, the expression of ADAM-33 was reported to be correlated to disease severity. Indeed, severe asthmatics display higher levels of ADAM-33 expression in their bronchial biopsies when compared to mild asthmatics or controls. Moreover, these asthmatics exhibit ADAM-33 staining in epithelial, submucosal and smooth muscle cells as demonstrated by immunohistochemistry [57]. This overexpression of ADAM-33 in the airways of asthmatics was also confirmed in animal models. Indeed, ADAM-33 levels were reported to increase in lungs of mice after allergen exposure [71]. Nevertheless, the demonstration of ADAM-33 implication in pathological processes leading to an asthma phenotype is still not fully accomplished. Indeed, phenotypes obtained in ADAM-33 KO mice did not suggest that the absence of ADAM-33 actually modulates baseline or allergen-induced airway responsiveness [72].

**Table 3:** ADAM-33 polymorphism studies in human populations.

Type of study	Population	Linkage asthma	Studied polymorphisms	Reference
FBAS; CC; LDT	UK Caucasian	yes	135	[53]
	US Caucasian			

FBAS; CC	Hispanic	no	6	[68]

CC	African from US	yes	8	[65]
	White from US			
	Hispanic from US			
	Dutch white			

FBAS; CC	German	yes	15	[64]

CC	Korean	yes	5	[89]

FBAS	North American *	no	17	[90]

LDT	Dutch Caucasian	yes	8	[91]

LDT	UK	yes	17	[92]

CC	German white *	no	10	[93]

CC	Japanese	yes	14	[66]

FBAS	Japenese *	yes	23	[63]

CC	Australian Caucasian	yes	10	[94]

CC	Northeast Chinese	no	3	[67]

FBAS	European-American *	no	ND	[95]
	hispanic *	no	ND	[95]

allele frequency	Japenese	yes	10	[96]

CC	Northeast Chinese	yes	6	[97]

allele frequency	Thai	yes	8	[98]

CC	Northeast Chinese	yes	6	[99]

FBAS; CC	UK	yes	4	[100]

Family- based association study: FBAS; Case-control study: CC; linkage disequilibrium test: LDT; ND: no determined; *: children

ADAM-8 is another member of the ADAM family potentially associated to asthma. The first report to suggest an ADAM-8 implication in asthma was published in 2004 [59]. This microarray study has shown that ADAM-8 expression is increased in mice exposed to allergens [59]. In 2008, another microarray study has confirmed the involvement of ADAM-8 in an acute model of asthma, mimicking the inflammation found in human airways, while no difference was found in the chronic model of asthma mimicking human airway remodeling [73]. Moreover, ADAM-8 mRNA levels are increased in sputum cells from asthmatic patients when compared to healthy subjects [51]. An immunohistochemistry targeting ADAM-8 has shown an elevated production of this proteinase in bronchial biopsies from asthmatics related to disease severity as reported for ADAM-33 [57]. A genomic study has recently reported a link between ADAM-8 single nucleotide polymorphisms and asthma in humans [74]. As membrane-bound CD23 is processed by ADAM-8 leading to ectodomain cleavage and resulting in the release of a soluble form of CD23 (sCD23), the low-affinity IgE receptor, ADAM-8 could take part to the cascade of events leading to asthma phenotype [51]. ADAM-8 has already been described to be a sheddase for CD23 [18,75]. The proteolytic release of CD23 from cells is likely to be a key event in allergic asthma. ADAM-8 also cleaves important effectors in asthma pathology such as pro-TNF-α and L-selectin [75,76]. Moreover, ADAM-8 is involved in macrophages activation [75]. The pharmacological delivery of IL-4 or IL-13 as well as use of mice transgene overexpressing these interleukins enhance ADAM-8 levels when mice are exposed to allergens suggesting that ADAM-8 depends not only from allergens but also from Th_2 _cytokines [59]. Other authors have studied the effects of an overexpression of a soluble form of ADAM-8 by liver tissue and did not find any difference regarding asthma phenotype [60]. Recently, we demonstrated that ADAM-8 is overexpressed in lungs from mice experimentally exposed to allergens and that the depletion of ADAM-8 by the use of KO animals or by immunodepletion dramatically decrease airway inflammation after allergen exposure (*Paulissen et al, submitted*). It is also worth noting that these ADAM-8 depleted animals do not display developmental abnormalities as described by Kelly *et *al [77]. Taken together, these data strongly suggest that ADAM-8 is a key mediator in asthma. Further studies should be performed in order to unveil the exact mechanisms implicating ADAM-8 in this disease.

Besides ADAM-8 overexpression, a modulation of RNA levels of ADAM-9, ADAM-12, ADAMTS-1 and ADAMTS-15 has been demonstrated in induced sputum from asthmatic patients [51]. Recently, a genomic study has demonstrated that many ADAM and ADAMTS proteinases such as ADAM-10,-17,-28 and ADAMTS-4, -9,-15 are also overexpressed in chronic asthma [73]. However, further studies might be led to explore their potential role in asthma-related pathology.

All these data highlight the implication of ADAM proteinases in asthma pathogenesis and suggest that new therapeutic strategies based on the inhibition of certain members of this proteinases family could be investigated.

## Contribution of ADAM and ADAMTS proteinases in COPD

**C**hronic **o**bstructive **p**ulmonary **d**isease (COPD) is characterized by a destruction of the lung parenchyma leading to alveolar wall destruction (emphysema) and important structural alterations in bronchial walls such as epithelial metaplasia or airway wall fibrosis [4]. The major risk factor for COPD is the inhalation of cigarette smoke. Despite the improvement of therapeutic strategies and a better understanding of this disease, the morbidity and mortality related to COPD are still significant. Matrix metalloproteinases such as MMP-9 and MMP-12 which have been reported to be modulated in airway secretions from COPD patients might contribute to disease progression and exacerbations by their catalytic activity. However, despite their potential importance in this disease, only few data are available concerning ADAM proteinases involvement in COPD (see figure 2).

ADAM-33 has also been identified as a susceptibility gene for COPD since **s**ingle **n**ucleotide **p**olymorphisms (SNPs) observed in this gene are associated with a higher risk for developing COPD [78]. ADAM-33 has recently been reported to be linked to airway hyperresponsiveness and airway inflammation in the general population suffering from COPD [79].

Data describing higher ADAM-17 (**TACE** for TNF-alpha converting enzyme) production in lung tissues from rats exposed to tobacco in a COPD model as compared to control animals support the implication of ADAM proteinases in this obstructive lung pathology [80]. Moreover, siRNA (small interfering RNA) raised against ADAM-17 mRNA as well as metalloproteinase inhibitors (GM-6001 and TNF-alpha inhibitor 1), prevent smoking- induced mucin overproduction in human airway epithelial cells (NCI-H292 cells) [81].

## Conclusions

Many peptidic mediators secreted in the lung by both structural as well as inflammatory cells are implicated in physiological processes and their overexpression or inhibition is in many cases part of intrinsic pathological mechanisms. ADAMs and ADAMTS proteins can cleave many of these factors and are therefore key mediators for the control of many biological processes in the lung. Among other activities, these proteinases are also active in the control of extracellular matrix homeostasis and cell migration. It seems therefore logical to set up some therapeutic strategies to target ADAM(TS) enzymes activity in obstructive airways diseases.

This review, aiming at summarizing some lung-related biological actions of ADAMs/ADAMTS, demonstrates to which extent these factors are important in both physiological and pathological processes in lung tissues. Many basic researches have still to be performed to clearly identify *target *proteinases that appear to play a direct role in pathogenesis as well as potential *anti-target *ADAMs whose inhibition could cause damages because they have a direct or indirect beneficial effect on lung physiology.

## Competing interests

The authors declare that they have no competing interests.

## Authors' contributions

GP drafted the manuscript. NR supervised the analysis of data and revised the manuscript. MMG, CC, FQC, SB, JH and MEH approved the final version of the manuscript. J-MF initiated the project. AN revised the manuscript critically. DDC initiated the project, was responsible to find grants, and approved the final version of the manuscript. All authors read and approved the final manuscript.
